# A curious formulation robot enables the discovery of a novel protocell behavior

**DOI:** 10.1126/sciadv.aay4237

**Published:** 2020-01-31

**Authors:** Jonathan Grizou, Laurie J. Points, Abhishek Sharma, Leroy Cronin

**Affiliations:** School of Chemistry, University of Glasgow, Joseph Black Building, University Avenue, Glasgow G12 8QQ, UK.

## Abstract

We describe a chemical robotic assistant equipped with a curiosity algorithm (CA) that can efficiently explore the states a complex chemical system can exhibit. The CA-robot is designed to explore formulations in an open-ended way with no explicit optimization target. By applying the CA-robot to the study of self-propelling multicomponent oil-in-water protocell droplets, we are able to observe an order of magnitude more variety in droplet behaviors than possible with a random parameter search and given the same budget. We demonstrate that the CA-robot enabled the observation of a sudden and highly specific response of droplets to slight temperature changes. Six modes of self-propelled droplet motion were identified and classified using a time-temperature phase diagram and probed using a variety of techniques including NMR. This work illustrates how CAs can make better use of a limited experimental budget and significantly increase the rate of unpredictable observations, leading to new discoveries with potential applications in formulation chemistry.

## Teaser

A bespoke robotic system driven by a curiosity algorithm discovers emergent behaviors of oil-in-water protocell formulations.

## INTRODUCTION

The investigation of multicomponent chemical formulation is a laborious and time-consuming effort. The combinatorial explosion, nonlinear properties, and rare events mean that even an expert experimentalist requires enormous resources to make significant discoveries. Although laboratory automation has shown a remarkable increase in experimental throughput (*1*, *2*), it does not change the relative rate of discoveries (with respect to the rate at which experiments are done) because the paradigm used to select experiments does not change alongside it. An appealing alternative is to implement the curious and knowledge-based inquiry process inherent in scientific researchers within a reliable and high-throughput robotic system (*3*–*5*). Statistical methods were previously introduced to analyze the vast quantities of data generated by laboratory robots (*6*, *7*), and recently, machine learning algorithms have started to be integrated into laboratory equipment (*8*, *9*). However, most of these methods focus on the optimization of targeted properties (*10*, *11*) or require previous knowledge (*12*, *13*).

**Fig. 1:**
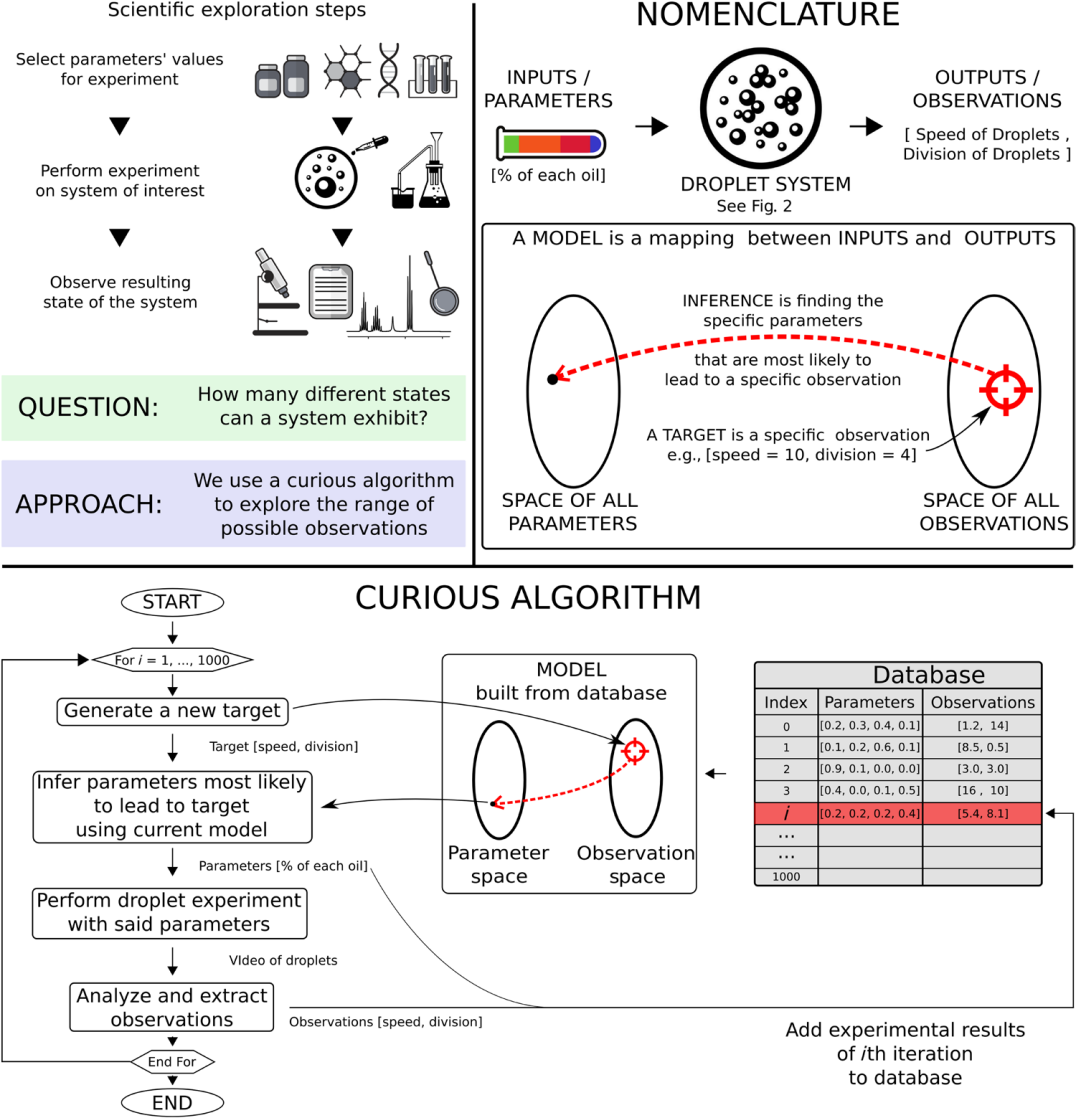
Description of the CA and the exploration methodology. (**Top**) Left: Explanation of the research question and approach. Right: Nomenclature of the terms used to describe our methodology using the droplet system in study as our example. (**Bottom**) Flow chart of the CA algorithm. At each iteration (one iteration = one experiment), the CA first generates a new temporary target that represents a desired observation. It then collates all the experimental results collected so far and uses them to build a model, which is used to infer the experimental parameters most likely to achieve the temporary target. The said experiment is then tested, and the results are stored in the dataset. The CA repeats this process until the budget allocated to the exploration is used up (1000 experiments in this work).

**Fig. 2:**
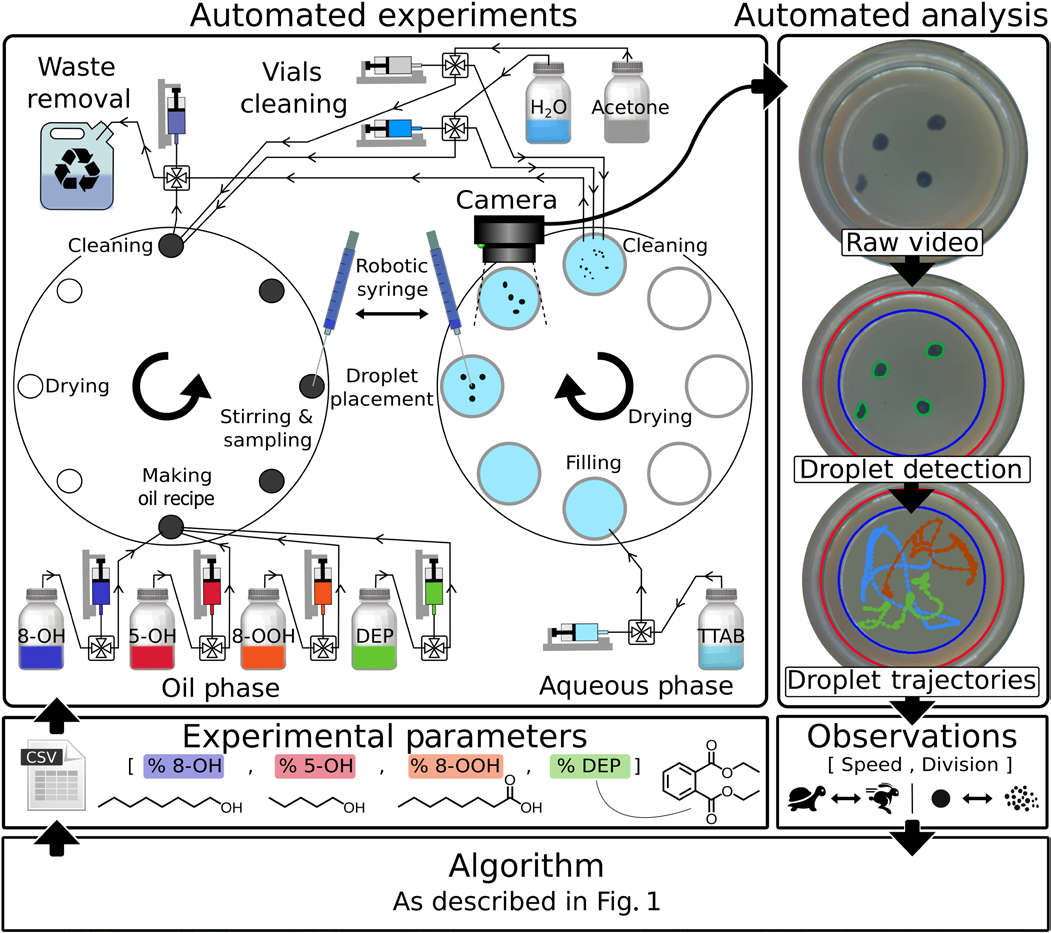
Diagram of the closed-loop workflow of the robotic platform. (**Top**) Left: Schematic of new high-throughput droplet-generating robot developed for this work in the “Robotic platform: Dropfactory” section in the Supplementary Materials. The robot runs the experiments by first mixing the oils according to specification and then prepares the aqueous phase and places droplets in the petri dish using a syringe. The motion of the droplet video is recorded and analyzed. Once the experiment is completed, the platform cleans the entire system. Right: Droplet contours and positions are extracted from the video data. (**Middle**) Right: From the trajectories, the average speed and number of droplets generated per experiment was determined. Left: Experimental parameters are the proportions of each oil comprising our droplets, which are then used by the platform to perform the next experiment. (**Bottom**) The CA learns from the observations and defines new experiments to be tested (see Fig. 1).

Here, we focus on exploration for its own sake. We describe an experimental method (Fig. 1) that implements state-of-the-art curiosity algorithms (CAs) into a newly designed parallel laboratory robot (CA-robot; Fig. 2 and see the “Robotic platform: Dropfactory” section in the Supplementary Materials). CAs have been developed to replicate curiosity-driven learning in humans (*14*, *15*) and make use of knowledge acquired from developmental psychology, neuroscience, artificial intelligence, and robotics (*16*). CAs have previously been shown very efficient at exploring systems in simulated problems or constrained robotic scenarios (*17*–*19*). Because CAs are designed to actively and autonomously select experiments that maximize the number of new and reproducible observations, applying CAs to the exploration of chemical systems could markedly improve the rate of new scientific observations in the laboratories.

Our CA, called random goal exploration (*17*, *20*), is the simplest of its algorithmic family, is easy to describe, and still performs comparatively to other implementations (*17*), making it an ideal candidate for this interdisciplinary didactic study. To select a new experiment, rather than deciding directly on experimental parameters, the CA generates a self-determined temporary target defined on the observation space.

The observation space is defined by the scientists and consists of properties of interest of the chemical system they wish to explore. The temporary target represents an observation that the CA-robot will try to generate from the chemical system by defining a new experiment. To do so, the CA-robot refers to the dataset of previous experiments performed and builds a temporary model of the system using a regression algorithm. The model is used to infer the experimental parameters that are most likely to lead to the observation of the self-determined target.

The selected experiment is then undertaken, leading to a new observation. The experiment results (both parameters and observations) are added to the dataset of previous experiments and will help improve the quality of the model, in turn improving the performance of the CA. The CA-robot repeats this process for a given number of iterations defined by the experimental budget allocated to the project. We highlight that the CA-robot always starts with zero experimental data and builds the dataset at the same time as it explores and learns about the system. For the first experiment, the CA cannot use any previous information. To define the *N*th experiment, it will be able to reuse the *N* − 1 experiments previously performed. We emphasize that a CA generates a new temporary target for each new experiment, and our CA samples temporary targets from a uniform probability distribution over the observation space. A detailed description of the algorithm is available in the “Curious algorithm: Random goal exploration” section in the Supplementary Materials.

To understand the benefit of this approach, consider the analogy with learning to play golf for the first time with no tuition. With each shot, you can vary how you hit the ball and with what club (your experimental parameters). Your aim is to learn a wide skill set and discover where you can send the ball (your observation space). Every time you play a shot, you learn from how it went and apply that knowledge to your future shots (you are building a model from the dataset of past experiences). The exploration question we consider in this work is: How do you allocate your time? Should you try contracting your muscles randomly and observe where the ball lands (random parameter search) or should you try to set yourself random targets to reach and observe how far from these targets the ball lands (our CA, called random goal exploration)? The problem is the same in experimental sciences: When faced with the task to decipher an unfamiliar system (hitting a ball with a club), should we try experiments at random and observe how the system reacts (contract your muscle randomly) or should we try to target specific states or properties and observe if we can generate them (set yourself different targets and learn from the process)? In the first approach (called “random” in this work), many experiments will tend to produce no interesting or new effects (e.g., missing the ball), and in the second approach (called “CA” in this work), many targeted states will tend to be out of reach of the system (e.g., putting the ball on the moon). However, the strength of the CA approach is that, even if many targets cannot physically be attained, the process of trying to reach them has been shown to generate more varied observations than the random approach and without the need of understanding the system in study (*17*).

We tested our approach on dynamic oil-in-water droplets—promising protocell models (*21*, *22*) displaying an astonishing range of lifelike behaviors, including movement, division, fusion, and chemotaxis (*23*–*26*). Although these droplets are thought to be driven by Marangoni instabilities originating from surface tension asymmetry (*27*), to date, the understanding of even the most simple systems remains limited (*28*, *29*). Hence, oil-in-water droplets offer a great example of the challenges in studying complex and poorly understood systems where few components can lead to the emergence of a range of complex properties or behaviors, a topic of great relevance across many industries. To perform the experiment, we designed a new high-throughput droplet dispensing robot with parallel operations (Fig. 2 and movie S1; see the “Robotic platform: Dropfactory” section in the Supplementary Materials) that can complete more than 30 experiments per hour, a six-time throughput increase from previously reported platforms (*24*, *26*). Our CA-robot can perform droplet experiments, record and analyze the droplets’ behaviors, and select the next experiments in full closed-loop autonomy.

## RESULTS

### Discovery of an anomaly

The first objective of this study was to compare the efficiency of our CA approach and a random parameter search (also called screening in high-throughput automation) at generating varied observations from our droplet system. We gave ourselves a finite experimental budget of 1000 experiments and compared the range of behaviors we could observe using the CA or the random algorithm—both algorithms being tested three times. Our parameter space is composed of all possible mixtures of four oils [octanoic acid, diethyl phthalate (DEP), 1-octanol, and 1-pentanol] from which our droplets are made. We chose our observation space as the droplet’s speed and number of divisions, both selected due to their inherently interesting nature and similarity to the behaviors of simple life-forms that can move and replicate (see the “Algorithms implementation” section in the Supplementary Materials for more details).

While these specific droplet behavioral metrics were relevant in this context, the methodology and principles applied here are not specific and could apply to many other metrics or systems. For example, we could consider the droplets’ shape as an additional dimension of observation. In reaction experiments, the parameters could be the quantity of each starting material and the environmental conditions (temperature, pressure, etc.), and the observation space could be the yield of each compound in the final product. In formulation experiments, the observation space could be the viscosity, density, elasticity, smell, color, etc. of a mixture that one might want to explore according to an initial mixture composition (the parameter space). Concretely, in pigment mixing experiments, the parameter space could be the composition of a mixture of pigments, and the observation space could be the resulting color after mixing, e.g., in the red-green-blue space.

To our surprise, during our first set of CA experiments, we noticed a marked change in the observable outputs for our third repeat compared with the first and second repeats, namely, at the third repeat, no droplets were observed with speed above 5 mm s^−1^ (see the “Observations leading to the discovery of a temperature effect” section in the Supplementary Materials). Our expectation was to get roughly the same range of droplet behaviors at each repeat because we considered the same droplet system and the same algorithm. After careful investigation of all possible causes for this anomaly (change in chemicals, experimental conditions, robotic process, tracking algorithm, etc.), we identified temperature as the most probable factor behind the observed phenomenon. The temperature in the room might have changed between the second and the third repeat. However, as in all previous reported work on this droplet system (*24*, *26*), the temperature was neither recorded nor controlled, and all experiments were performed at room temperature. A new set of questions emerged: (i) Can a change of only a few degrees Celsius really affect our droplet system? If yes, how and to what extent? (ii) Was it the CA algorithm that allowed the observation of this anomaly? Or would it have been as likely for us to make our serendipitous observation with the random algorithm if the temperature had changed too? We answer first the latter questions and then characterize thoroughly the temperature effect on our droplet system.

### Proving that the discovery was enabled by the CA algorithm

**Fig. 3:**
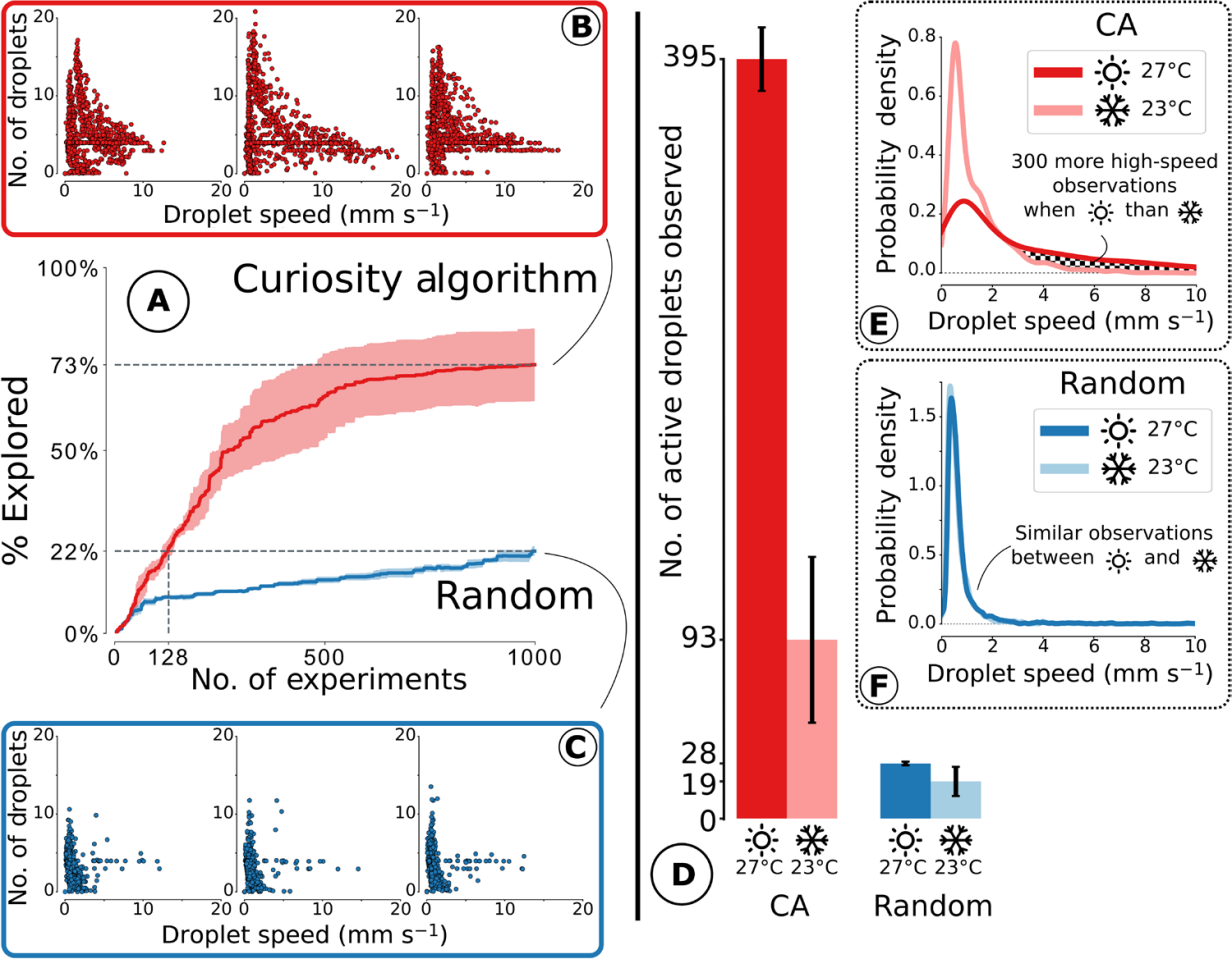
A summary of the results generated using our CA-robot, illustrating how the CA enables both significantly greater exploration of the behavioral space and the discovery of temperature sensitivity of the droplets. Left: Comparison of the observed droplet behaviors after 1000 individual experiments for CA and random (average of three repeats, with shaded area showing 68% confidence interval). (**A**) Evolution of the percentage of the behavior space explored between the two methods. CA explored 3.3 times more within the same experimental budget (73% versus 22%) and generated as diverse observations as random after only 128 experiments—a sevenfold reduction in time and financial cost for equivalent results. (**B** and **C**) Visualization of the observations made by each method for each repeat; each scatter dot represents the average speed and number of droplets for a single 90-s droplet experiment. CA (B) leads to many more observations of rare and interesting droplets than random (C). Right: Effect of temperature (22.6° ± 0.5°C versus 27.0° ± 0.7°C) on the observations made using each algorithm. (**D**) Number of droplet experiments observed with a speed faster than 3 mm s^−1^ for each method and temperature, with error bars showing the SD. The CA-robot, by performing the same number of experiments, generated 14 times more interesting droplet recipes than random at 27.0°C (395 versus 28, *P* < 0.001) and 5 times more at 22.6°C (93 versus 19, *P* = 0.13). A change of only ca. 4.4°C led to a large and significant difference in the observed droplet behaviors when using the CA (395 versus 93, *P* = 0.005). This difference in effect could not be significantly observed when using random (28 versus 19, *P* = 0.22). This is confirmed by (**E**) and (**F**), which show the distribution of observation for CA and random, respectively. (E) The distribution of observations has a strong tail indicating a wider exploration from the CA-robot, and there is a significant difference between observations made at 27.0° and 22.6°C that is not observable at random (F). By focusing on the output space, the CA-robot provides a more accurate picture of the system for the same experimental budget, which allowed the discovery of this delicate temperature effect.

To test whether our discovery was enabled by the CA algorithm, we ran three repeats of both algorithms (CA and random) at 22.6° ± 0.5°C and 27.0° ± 0.7°C (mean ± SD). At 27°C, and given the same budget of 1000 experiments (each lasting 90 s), the CA-robot generated significantly more varied droplet behaviors than the random parameter search (Fig. 3, B and C; notice the higher speed and division of droplets observed using the CA versus the random methodology). We quantified this exploration (see the “Exploration measure” section in the Supplementary Materials) and found that the CA enables us to observe 73.4 ± 15.2% of the total observable space, ca. 3.3× more (*P* = 0.039, Welch’s *t* test) than a random parameter search (22.5 ± 2.1%) within the same experimental budget. After only 128 experiments, the CA-robot already generated more varied experiments than random parameter search did in 1000 experiments (Fig. 3A), a sevenfold efficiency gain in time and resources given the same hardware setup. Movie S2 illustrates the exploration over time using both the CA and random; notice how even after as few as 50 experiments, the CA-driven exploration is already identifying more extreme cases of droplet behavior, and this differentiation only increases as more experiments are undertaken. Strikingly, the number of active droplet experiments observed (speed, >3 mm s^−1^) is as low as 28.7 ± 0.9 for random parameter search but jumps to 395.0 ± 16.5 for the CA, a 14-fold improvement (*P* < 0.001), without explicitly asking the robot to generate high-speed experiments. This is further visualized in movie S3, which shows videos of the 1st, 10th, and 50th highest speed recipes from the two approaches.

The above result shows convincingly that, at a given temperature of 27°C and with a given budget of 1000 experiments, the CA enables us to observe more varied droplet behaviors than a random parameter search. But could the temperature effect still have been observed by the random parameter search? Figure 3 compares the distribution of the speed of droplet experiments generated by both algorithms at 22.6° ± 0.5°C and 27.0° ± 0.7°C. The ca. 4.4°C temperature change has a significant impact on the observations made using the CA (395.0 ± 16.5 versus 93 ± 43.1 active droplets, *P* = 0.005), while a negligible change is observed with a random parameter search (28.7 ± 0.9 versus 19.3 ± 7.6 active droplets, *P* = 0.22). Notice the differences in the distribution of speed observed for each algorithm at both temperatures in Fig. 3 (E and F). This key result allows us to claim that our initial observation of the temperature “anomaly” was only feasible because of the exploratory benefits that our CA algorithm provides. By extension, we have shown that to explore a new system along properties of interest, it is more efficient to set temporary targets randomly in the observation space and to try to reach them than to try random combinations of parameters. In other words, using a CA over a random parameter search to design exploratory experiments for an unfamiliar system is a better use of a limited experimental budget.

### Characterizing the temperature effect

**Fig. 4:**
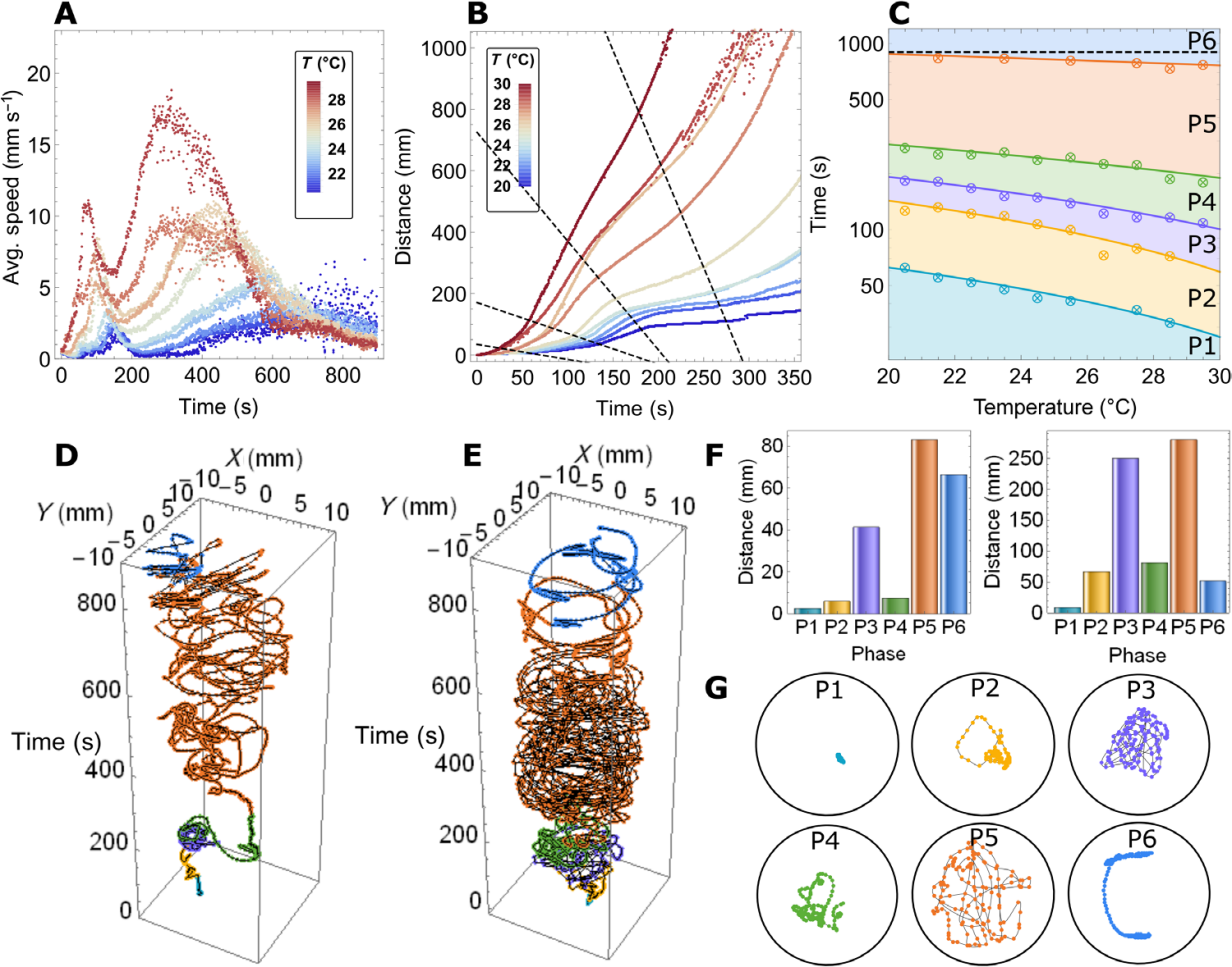
A summary of the analysis undertaken on a focus recipe, which resulted in the classification of six phases of droplet motion and the production of a time-temperature phase diagram. (**A**) Temperature dependence of droplet speed versus time. Each color represents all experiments consisting of four droplets undertaken in a given temperature interval of 1°C. (**B**) Temperature dependence of droplet cumulated distance moved versus time. The black dashed lines show the phase transitions in droplet motion that are used to estimate the phase diagram and are calculated by linear fitting of maxima and minima in the acceleration profile at each temperature interval. (**C**) Temperature-time phase diagram of droplet motion showing different phases: initiation (P1), fluctuation (P2), irregular (P3), deceleration (P4), continuous (P5), and saturation (P6). The marked data points correspond to the intercepts shown in (B). (**D** and **E**) The trajectory of a single droplet at 21.44°C (D) and 27.39°C (E), with different motion phases highlighted by color. (**G**) Exemplar 36-s segments of each phase of motion, with each point showing the droplet location every 0.25 s at 27.39°C (E). Each example trajectory contains the same number of points to emphasize the differences in distance covered during the different phases, which is quantified in the cumulative distance per phase plots (**F**) for the droplet trajectories seen in (D, left) and (E, right).

To study this newly observed effect in detail, we ran targeted droplet experiments within the range of temperatures accessible in the room (20° to 30°C). There were significant, unexpected, and nonlinear variations in the behavior of the droplets of different compositions due to temperature (see the “25 recipe temperature screen” section in the Supplementary Materials). Such variations were highly reproducible, as, for a given recipe, the observation of droplets’ behavior is enough to infer the room temperature with high accuracy (prediction error of 0.05 ± 0.66°C; see the “Droplets as temperature sensors” section in the Supplementary Materials), a testament to both the reproducibility of the droplet behaviors and the existence of a delicate temperature effect. This is rather striking given the complexity of the system, the time scale of an experiment, and the relative simplicity of our video-based analysis. One recipe of interest (composed of 1.9% octanoic acid, 47.9% DEP, 13.5% 1-octanol, and 36.7% 1-pentanol) was further analyzed. The vast differences of speed observed with this recipe to small temperature changes are illustrated in movie S4. To probe the causes behind these observations, we ran longer (15-min) droplet experiments at a range of temperatures (see the “15 minute experiments” section in the Supplementary Materials). Surprisingly, as shown in Fig. 4A and movie S5, the droplets were seen to exhibit two peaks in their speed-time profile—they accelerate to achieve a first maximum speed, decelerate, and then accelerate again to reach a second maximum speed. The temperature effect on droplet motion can be seen in the variation of their speed profile, with the peak speed timing and magnitude exhibiting clear trends with temperature, with the peaks occurring earlier and with a greater magnitude for hotter experiments.

Using the droplet displacement data, we identified six clear stages of droplet motion: initiation, fluctuation, irregular, deceleration, continuous, and saturation, of which characteristic examples may be seen in Fig. 4G (P1 to P6). During the initiation stage, the droplet vibrates around a point, showing little locomotion and low speeds. During fluctuation, these vibrations extend and the droplet speed increases before peaking during irregular motion, in which the droplet moves short distances in alternating directions. This is followed by a deceleration stage, during which the droplets slow down and display smoother motion, which then develops into continuous motion, during which concerted movement is seen and resulting in a more circular motion of the droplets around the dish. Eventually, the saturation stage is reached, in which the droplets slow down again and come to a halt. The peak speeds are observed for the irregular (purple) and continuous (orange) modes of motion, with the deceleration (green) period existing in between these two. A temperature-time phase diagram was derived showing the times at which each distinct phase of motion occurs at different temperatures (Fig. 4C and see the “Generating the temperature-time phase diagram” section in the Supplementary Materials). The temperature-time phase diagram was created by calculating the intercept between cumulative distance traveled plots and linearly fitted transition times (Fig. 4B). The phase transition times were each defined by characteristic points in the droplet acceleration time plots. This phase diagram highlights the strong temperature dependence on the duration of each of the phases of motion and can be used to predict the mode of droplet motion observed at any time or temperature within the studied range.

**Fig. 5:**
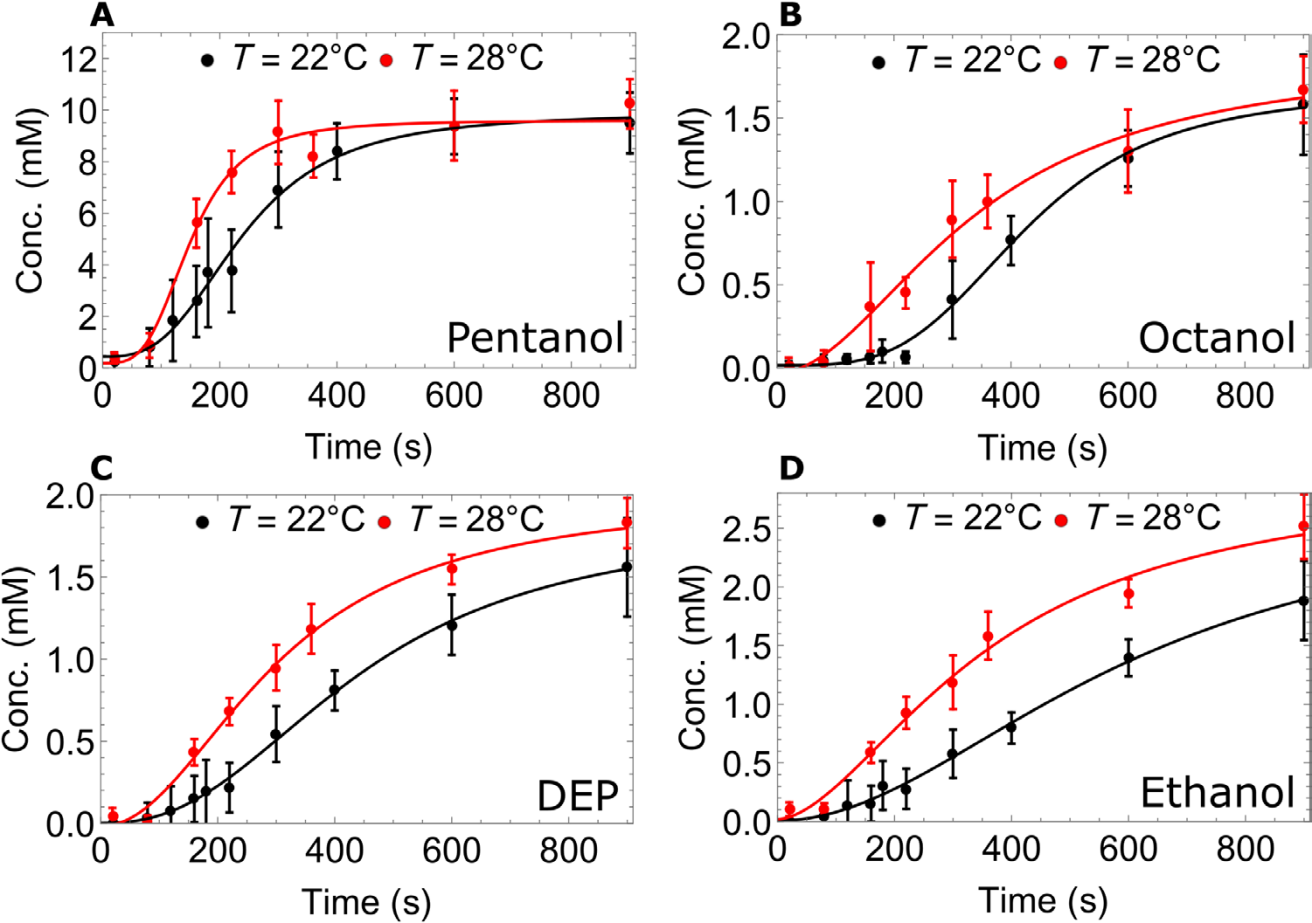
Oil concentration in the aqueous phase over time at 22°C (black) and 28°C (red), as quantified by ^1^H NMR spectroscopy. Note how each oil (**A** to **D**) dissolves faster at the higher temperature, while DEP (C) and ethanol (D) also dissolve to different final concentrations. Note differences in *y*-axis scale—pentanol (A) dissolves around five as much as the other oils. When regulated to a target of 22°C, the temperature at the experimental location was 22.4° ± 0.2°C. When regulated to a target of 28°C, the temperature at the experimental location was 27.7° ± 0.2°C.

Oil dissolution into the aqueous phase is hypothesized to play a major role in the observed droplet behaviors (*27*, *29*), with oil dissolution affecting the interfacial tension, leading to droplet motion induced by Marangoni instabilities. We used a previously reported ^1^H nuclear magnetic resonance (NMR) spectroscopic method (*24*) to quantify the aqueous phase oil concentration during droplet motion at 22.4° ± 0.2°C and 27.7° ± 0.2°C (see the “^1^H NMR oil dissolution analysis” section in the Supplementary Materials). A 5°C temperature increase is seen to accelerate the dissolution of all oils (Fig. 5).

Pentanol dissolves fastest and to the greatest level, as expected by its relative solubility. Octanoic acid dissolves to a fixed level early in the experiment and then stays constant; this is expected due to its low concentration in the formulation and the fact that it will rapidly deprotonate at high pH. As previously reported (*24*), we note the presence of ethanol due to the base catalyzed hydrolysis of DEP. DEP and ethanol have different final concentrations at the different temperatures, as temperature affects the equilibrium of the hydrolysis reaction, as opposed to only physical processes driving the other oil dissolution. Octanol, DEP, and ethanol dissolution are delayed as compared to pentanol dissolution, suggesting that pentanol dissolution is the main contributor to the first peak of droplet motion.

**Fig. 6:**
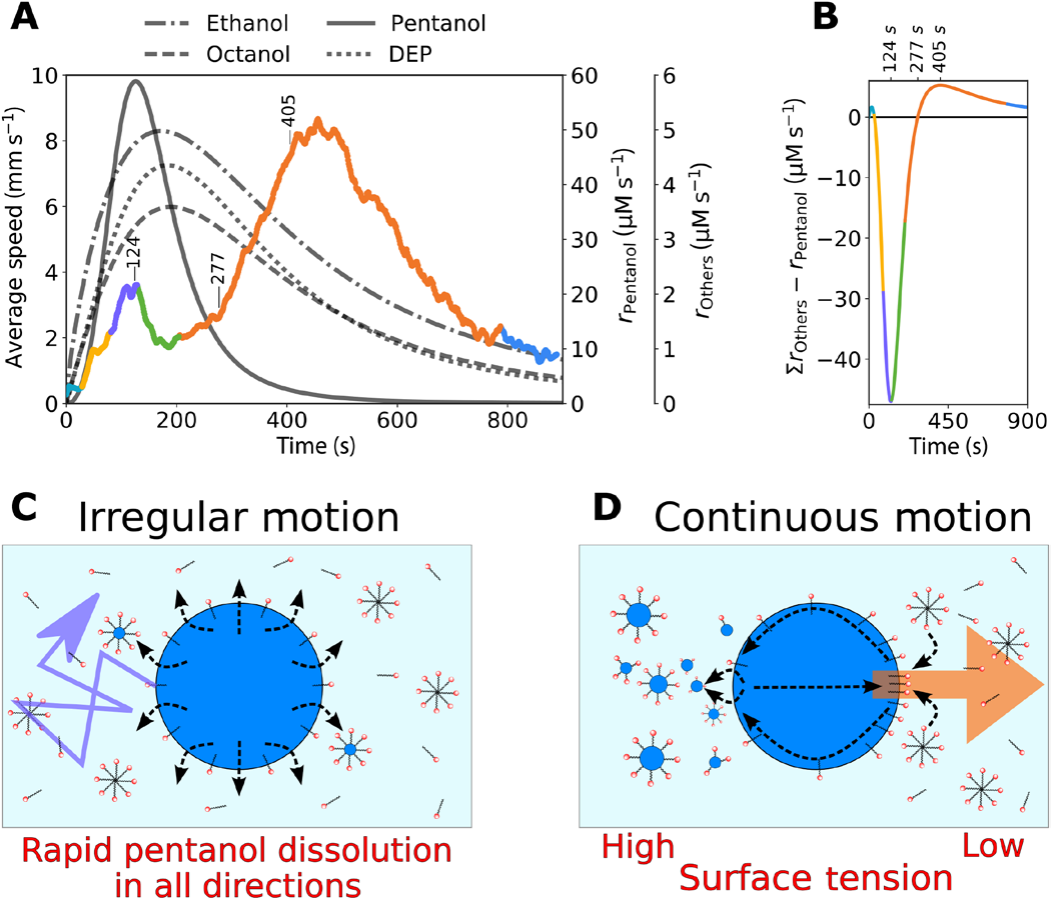
The correlation between oil dissolution and droplet behaviors and schematics illustrating the proposed mechanisms for the irregular and continuous phases of motion. (**A**) Average oil droplet speed (colored plot; left, *y* axis) observed at 28°C (average across eight experiments processed via a 10-s moving average), with the color corresponding to the phase of motion (cyan, initiation; yellow, fluctuation; purple, irregular; green, deceleration; orange, continuous; and blue, saturation). The gray lines illustrate the rates of oil dissolution (right hand, *y* axes) from the fitted ^1^H NMR spectroscopy dissolution data. (**B**) Difference between the sum of the rates of DEP, ethanol, and octanol dissolution and the rate of pentanol dissolution against time. Note the peak difference in favor of pentanol at 124 s, the point at which the rates are equal at 277 s and the peak difference in favor of DEP, ethanol, and octanol at 405 s. These times are also marked in (A) and correlate closely with the irregular-deceleration transition, rapid acceleration in the continuous phase, and the maximum droplet speeds in the continuous phase. (**C**) Schematic illustrating the proposed mechanism for the fluctuation and irregular phases of motion. Rapid pentanol dissolution in all directions (black arrows) into a largely oil free aqueous phase containing many empty micelles and free surfactants leads to no concerted directional motion but rather erratic motion in various directions (purple arrow). (**D**) Schematic illustrating the proposed mechanism for the continuous phase of motion. At this time, total oil dissolution is slower. The front of the moving droplets contacts “fresh” aqueous phase, while the rear of the droplet leaves a trail of “filled” micelles. Thus, the interfacial tension is lower at the front of the droplet, leading to a positive feedback loop of forward motion via Marangoni flows.

To confirm this hypothesis, we compared the oil dissolution rates with the droplet motion data, as shown in Fig. 6 (A and B) and detailed in the “Associating physical and chemical analysis” section in the Supplementary Materials. The rate of pentanol dissolution is seen to be rapidly increasing during the fluctuation and irregular phases before rapidly decreasing during the deceleration phase. This indicates that pentanol dominates the early stages of droplet motion and that its dissolution is the primary cause of the fluctuation and irregular forms of motion. As pentanol dissolves so fast in these early stages, it is expected that the motion is sporadic, as rapid dissolution in all directions (Fig. 6C) prevents the initiation of structure, regular flows, and a more continuous form of motion. Because pentanol dissolution has largely ceased by the time of the continuous phase of motion, while the other oils are still dissolving to significant levels, it appears that DEP/ethanol and/or octanol are the primary driving forces of the continuous period of motion. We hypothesize (Fig. 6D) that the more gradual rate of dissolution during the continuous phase of motion allows a positive feedback loop to be set up between oil motion, dissolution, and Marangoni flows (*30*, *31*). As the droplet moves in this phase, it advects “fresh” surfactant solution onto its anterior face (via collision with empty micelles and free surfactant molecules) and leaves a trail of oil-filled micelles in its wake (via oil dissolution). Thus, the interfacial tension is higher at the posterior face, as there are more oil-filled micelles and less free surfactants in this zone. As there is an interfacial tension differential between the anterior and posterior faces of the moving droplet, a Marangoni flow is induced, supporting the forward direction of motion, providing a positive feedback loop for continued forward motion. This hypothesis is also supported by the observation that droplets often avoid following the recent path of other droplets. When the oil dissolution rates begin to saturate, the continuous motion slows and stops.

We cannot ascertain from the previously discussed data whether it is DEP, ethanol, and/or octanol dissolution that is the primary cause of the continuous phase of motion. To discriminate between these, we varied the pH of the surfactant containing aqueous phase, which had a significant impact on the oil droplet behavior. As the pH and temperature are increased, DEP hydrolysis is significantly accelerated, (*32*), leading to an earlier and larger second continuous motion peak (fig. S36). With increasing pH, there is also a 10^6^-fold increase in ionic strength, significantly reducing the aqueous solubility of alcohols (*33*), thus lowering the dissolution of pentanol and reducing the irregular motion peak. These results together indicate that DEP hydrolysis is the primary cause of the second movement peak and continuous phase of motion. A range of experiments in which the pentanol-octanol ratio, the alcohol chain length, and the number of droplets placed were varied further confirmed the links between pentanol and the first speed peak and DEP and the second speed peak (see the “Additional experiments to probe the system” section in the Supplementary Materials).

As a proof of concept, we investigated the use of droplets as containers with temperature-dependent release for active molecules. Our experiment showed that the dye methylene blue was released 2.5 times faster at 28.6° ± 0.6°C than at 17.6° ± 0.2°C (see the “Temperature controlled dye release” section in the Supplementary Materials and movie S6).

## DISCUSSION

By designing a droplet-generating robot equipped with a CA (CA-robot), we were able to uncover the temperature sensitivity of our self-propelled droplet system. We demonstrated that, given the same experimental budget, this temperature effect could not have been observed using a random parameter search. This illustrates that CA-robots can be of significant advantage to assist scientists in revealing properties of unfamiliar systems as they can generate a wider variety of observation. Using physical and chemical analysis, we characterized the discovered effect and derived a phase diagram of droplet motion through time and temperature, which links to the underlying oil dissolution processes. This chemical analysis revealed the astonishing complexity that underlies the dynamics of our four-component oil-in-water droplet system. This is the first time a CA has been used for the exploration of a physical system in the laboratory using a fully automated robotic platform. Future research will focus on constructing the observation dimensions autonomously from the droplet videos in an unsupervised way, as in this work the observation space was designed by the authors, which potentially introduces human bias that can limit possible discoveries.

## MATERIALS AND METHODS

### Robotic platform

We designed a high-throughput droplet-generating robot (Fig. 2) that can execute and record a 90-s droplet experiment every 111 s, including mixing, syringe-driven droplet placement, recording, cleaning, and drying. This minimal overhead time was achieved by parallelizing all operations, enabling our platform to routinely perform 300 droplet experiments per day in full autonomy. The platform and sequence of operations are described in the “Robotic platform: Dropfactory” section in the Supplementary Materials, with code and design available online.

### Droplet chemistry

The oil-in-water system comprises four droplets composed of a mixture of four oils placed onto a surfactant containing aqueous phase in a petri dish (*26*). An experiment consists of preparing a formulation of octanoic acid, DEP, 1-octanol, and 1-pentanol at a specific ratio determined by the algorithm and dyed with Sudan Black B dye (0.5 mg ml^−1^). The oil mixture was sampled by the robot using a 250-μl syringe and delivered as 4 × 4 μl droplets in a Y pattern from the center of a 32-mm petri dish filled with 3.5 ml of a 20 mM cationic surfactant [myristyltrimethylammonium bromide (TTAB)] solution raised to a high pH (ca. 13) using NaOH (8 g liter^−1^). The droplet-making procedures are described in the “Oil and aqueous phase preparation” section in the Supplementary Materials.

### Image analysis

Droplet experiments lasted 90 s for exploration experiments and 900 s for the time-temperature phase diagram analysis. The droplet activity was recorded at 20 fps and analyzed using computer vision. Droplet contours were extracted using a thresholding algorithm and tracked through frames using a proximity rule. The droplets’ average speed and the average number of droplets in the dish (droplets can split, fuse, or leave the tracking area) were quantified and used as the observation space for exploration experiments. For time-temperature profiles, metrics were computed through time using a sliding window of 2 s. The droplet tracking procedures are described in the “Droplet tracking” section in the Supplementary Materials.

### Algorithmic implementation

Experimental parameters were generated as a four-dimensional vector representing the ratio of each oil in the droplet mixture. Observations were represented as a two-dimensional vector representing the average speed and average number of droplets in an experiment. The CA algorithm, called random goal exploration, samples temporary targets from a uniform distribution over the observation space. The forward model was built uniquely from previous observations using locally weighted linear regression, and the inverse model was solved for each target using the CMA-ES algorithm on the learnt forward model. The CA implementation is described in the “Algorithms implementation” section in the Supplementary Materials.

### Phase diagram experiments

To investigate the temperature effect, 72 15-min experiments were performed at temperature ranging from 20° to 30°C. Fifty-nine of them, evenly distributed in the temperature range, were exploited to build the time-temperature phase diagram. The experiment was binned in 1°C intervals, and phase changes were identified from inflection points in the droplet average speed and acceleration profile for each bin. The procedure is described in the “15 minute experiments” and “Generating the temperature-time phase diagram” sections in the Supplementary Materials.

### Data availability

Because of the large total size of the droplet videos (>500 gigabytes of data), the experimental data used in this work are available upon request to the corresponding author (lee.cronin@glasgow.ac.uk).

### Code availability

The code used to operate the robotic platform and generate and analyze results is available online in our group GitHub account at https://github.com/croningp and is described in the Supplementary Materials.

## ASSOCIATED CONTENT

### Supplementary information

The Supplementary Information Appendix contains further results and discussion including more details on related work, an in-depth comparison of the algorithms and a detailed explanation of the physicochemical analysis undertaken, the modeling of droplet behavior, and the phase diagram preparation. Additional experiments are presented studying the sensitivity of our system to pH, proportion of each oil, chain length of alcohol used and the number of droplets place in the dish, as well as detail given on the dye release and droplets as temperature sensor experiments. The Supplementary Information Appendix also provides detailed information (and the relevant GitHub repositories) about the materials and methods including the full droplet robot design and code, the droplet tracking implementation, a formal description of the CA and its implementation, and the experimental procedure related to the chemical analysis. Last, the supplementary movies are listed along with their explanatory captions.

## Supplementary Material

http://advances.sciencemag.org/cgi/content/full/6/5/eaay4237/DC1

Download PDF

Movie S1

Movie S2

Movie S3

Movie S4

Movie S5

Movie S6

A curious formulation robot enables the discovery of a novel protocell behavior

## SUPPLEMENTARY MATERIALS

Supplementary material for this article is available at http://advances.sciencemag.org/cgi/content/full/6/5/eaay4237/DC1 Supplementary Results and Discussion Supplementary Materials and Methods Fig. S1. Data leading to the discovery of an anomaly. Fig. S2. Temperature recordings for experiments performed at 27°C. Fig. S3. Observations by CA and random at 27°C. Fig. S4. Density of observations by CA and random at 27°C. Fig. S5. Density of observations by CA and random at 27°C with equal scale. Fig. S6. Distribution of observations by CA and random at 27°C. Fig. S7. Evolution of the exploration measure for CA and random at 27°C. Fig. S8. Observations by CA and random at 27°C every 100 iterations. Fig. S9. Distribution of parameters selected using the random algorithm at 27°C. Fig. S10. Distribution of targeted observations by the CA at 27°C. Fig. S11. Distribution of parameters selected by the CA at 27°C. Fig. S12. Distribution of the ratios of each oil explored by CA and random at 27°C. Fig. S13. Distribution of resulting formulation properties by CA and random at 27°C. Fig. S14. Distribution of droplet dynamical properties by CA and random at 27°C. Fig. S15. Distribution of droplet size by CA and random at 27°C. Fig. S16. Temperature recordings for experiments performed at 23°C and 27°C. Fig. S17. Comparison of observations by CA and random at 23°C and 27°C. Fig. S18. Comparison of density of observations by CA and random at 23°C and 27°C. Fig. S19. Comparison of exploration measure for CA and random at 23°C and 27°C. Fig. S20. Comparison of distribution of observations for CA and random at 23°C and 27°C. Fig. S21. Comparison of ratios of pentanol in droplet formulation for CA and random at 23°C and 27°C. Fig. S22. Experiments properties at a range of temperatures (17°C to 30°C) for 25 selected recipes. Fig. S23. Measured versus predicted temperature of 140 droplet experiments based uniquely on their video. Fig. S24. Recorded temperatures for the 20 repeats of the dye release experiments. Fig. S25. Histogram of the color change at the start and end of a dye release experiment. Fig. S26. Ratio of pixels dyed blue against time at 18°C and 29°C. Fig. S27. Evolution of droplet division and speed metrics during a single 15-min experiment. Fig. S28. Evolution of droplet division and speed metrics during a single 15-min experiment in the temperature range 20° to 30°C. Fig. S29. Workflow used in the preparation of the temperature-time phase diagram. Fig. S30. Cumulated distance traveled by droplets during a 15-min experiment. Fig. S31. Spread of temperatures of the 59 experiments used for phase diagram preparation. Fig. S32. Cumulated distance traveled by droplets binned in different temperature intervals. Fig. S33. Reconstructed speed and acceleration of droplets from the cumulative displacement data. Fig. S34. Temperature-time dependence on droplet behavior. Fig. S35. 3D visualization of a droplet trajectory in time and space at 21°C and 27°C. Fig. S36. Concentrations of oils in the aqueous phase through time. Fig. S37. Comparison between rates of oil dissolution estimated from NMR experiments and cumulated distance travelled. Fig. S38. Droplet speed evolution as temperature and aqueous phase pH are varied. Fig. S39. Dissolution level of each oil as temperature and aqueous phase pH are varied. Fig. S40. Impact of small changes in the proportion of each oil on the droplet speed-time profile during a 15-min experiment. Fig. S41. Same as fig. S40 with zoom on the first 200s. Fig. S42. Same as fig. S40 showing standard deviation. Fig. S43. Impact of replacing pentanol with oil of varied chain length on the droplet speed-time profile during a 15-min experiment. Fig. S44. Impact of number of droplet placed in the dish on the droplet speed-time profile during a 15-min experiment. Fig. S45. Same as fig. S44 with standard deviation. Fig. S46. Hydrodynamic diameter of micelles in the aqueous phase through time. Fig. S47. Conceptual design of the new Dropfactory laboratory robot. Fig. S48. Photo of the Dropfactory robot. Fig. S49. 3D view of the CAD design. Fig. S50. Geneva wheel design. Fig. S51. Geneva wheel top-plates design. Fig. S52. Photo of the Geneva wheel installed on Dropfactory. Fig. S53. Wheel stabilizer design and photo. Fig. S54. Modular linear actuator used designed for Dropfactory. Fig. S55. Photo of pumps and chemical inputs on Dropfactory. Fig. S56. Illustrating the working stations on the oil Geneva wheel. Fig. S57. Photo of the oil filling station. Fig. S58. Design of the oil filling head. Fig. S59. Photo of the oil stirring station with the small magnetic stirrer plate. Fig. S60. Photo and design of the oil cleaning station. Fig. S61. Photo of the oil drying stations. Fig. S62. Design of the drying station air guide. Fig. S63. Illustrating the working stations on the aqueous Geneva wheel. Fig. S64. Photo of the aqueous filling station. Fig. S65. Design of the aqueous filling station tube guide. Fig. S66. Photo of the syringe pick and place station. Fig. S67. Design of the modular syringe driver. Fig. S68. Photo of the recording station. Fig. S69. Design of the recording station. Fig. S70. Photo of the dish cleaning station. Fig. S71. Design of the dish cleaning station. Fig. S72. Photo of the drying station. Fig. S73. Visual display of droplet placement considered. Fig. S74. Comparison of biased droplet motion induced by droplet placement. Fig. S75. Droplet tracking via OpenCV. Fig. S76. Explanation of threshold definition for binarization of droplet video. Fig. S77. Image processing pipeline for the detection of droplet. Fig. S78. Visualization of the covered arena area metrics. Fig. S79. Visualization of the exploration metrics. Table S1. Description of each phases P1 to P6 with identifying criteria. Table S2. Parameters values for oil concentration curve fitting. Table S3. Measured PH of aqueous phase preparation. Movie S1. Operation of the parallelized droplet robot. Movie S2. Progression of the exploration for each algorithm. Movie S3. 1st, 10th, and 50th highest speed droplet recipes from each algorithm. Movie S4. Effect of temperature on a droplet recipe during a 90s experiment. Movie S5. Effect of temperature on a droplet recipe during a 15-min experiment. Movie S6. Effect of temperature on the release of methylene-blue dye. References (*34*–*56*)
