# Supplementary material for: A curious formulation robot enables the discovery of a novel protocell behavior
Source: Sci Adv. 2020 Jan 31;6(5):eaay4237. doi: 10.1126/sciadv.aay4237 (PMC6994213; doi:10.1126/sciadv.aay4237)
Supplement: http://advances.sciencemag.org/cgi/content/full/6/5/eaay4237/DC1 [file supp_6_5_eaay4237__index.html]

Science Advances | Science AdvancesAAASSearchScience AdvancesMenu

## Supplementary Materials

**The PDFset includes:**

- Supplementary Results and Discussion
- Supplementary Materials and Methods
- Fig. S1. Data leading to the discovery of an anomaly.
- Fig. S2. Temperature recordings for experiments performed at 27°C.
- Fig. S3. Observations by CA and random at 27°C.
- Fig. S4. Density of observations by CA and random at 27°C.
- Fig. S5. Density of observations by CA and random at 27°C with equal scale.
- Fig. S6. Distribution of observations by CA and random at 27°C.
- Fig. S7. Evolution of the exploration measure for CA and random at 27°C.
- Fig. S8. Observations by CA and random at 27°C every 100 iterations.
- Fig. S9. Distribution of parameters selected using the random algorithm at 27°C.
- Fig. S10. Distribution of targeted observations by the CA at 27°C.
- Fig. S11. Distribution of parameters selected by the CA at 27°C.
- Fig. S12. Distribution of the ratios of each oil explored by CA and random at 27°C.
- Fig. S13. Distribution of resulting formulation properties by CA and random at 27°C.
- Fig. S14. Distribution of droplet dynamical properties by CA and random at 27°C.
- Fig. S15. Distribution of droplet size by CA and random at 27°C.
- Fig. S16. Temperature recordings for experiments performed at 23°C and 27°C.
- Fig. S17. Comparison of observations by CA and random at 23°C and 27°C.
- Fig. S18. Comparison of density of observations by CA and random at 23°C and 27°C.
- Fig. S19. Comparison of exploration measure for CA and random at 23°C and 27°C.
- Fig. S20. Comparison of distribution of observations for CA and random at 23°C and 27°C.
- Fig. S21. Comparison of ratios of pentanol in droplet formulation for CA and random at 23°C and 27°C.
- Fig. S22. Experiments properties at a range of temperatures (17°C to 30°C) for 25 selected recipes.
- Fig. S23. Measured versus predicted temperature of 140 droplet experiments based uniquely on their video.
- Fig. S24. Recorded temperatures for the 20 repeats of the dye release experiments.
- Fig. S25. Histogram of the color change at the start and end of a dye release experiment.
- Fig. S26. Ratio of pixels dyed blue against time at 18°C and 29°C.
- Fig. S27. Evolution of droplet division and speed metrics during a single 15-min experiment.
- Fig. S28. Evolution of droplet division and speed metrics during a single 15-min experiment in the temperature range 20° to 30°C.
- Fig. S29. Workflow used in the preparation of the temperature-time phase diagram.
- Fig. S30. Cumulated distance traveled by droplets during a 15-min experiment.
- Fig. S31. Spread of temperatures of the 59 experiments used for phase diagram preparation.
- Fig. S32. Cumulated distance traveled by droplets binned in different temperature intervals.
- Fig. S33. Reconstructed speed and acceleration of droplets from the cumulative displacement data.
- Fig. S34. Temperature-time dependence on droplet behavior.
- Fig. S35. 3D visualization of a droplet trajectory in time and space at 21°C and 27°C.
- Fig. S36. Concentrations of oils in the aqueous phase through time.
- Fig. S37. Comparison between rates of oil dissolution estimated from NMR experiments and cumulated distance travelled.
- Fig. S38. Droplet speed evolution as temperature and aqueous phase pH are varied.
- Fig. S39. Dissolution level of each oil as temperature and aqueous phase pH are varied.
- Fig. S40. Impact of small changes in the proportion of each oil on the droplet speed-time profile during a 15-min experiment.
- Fig. S41. Same as fig. S40 with zoom on the first 200s.
- Fig. S42. Same as fig. S40 showing standard deviation.
- Fig. S43. Impact of replacing pentanol with oil of varied chain length on the droplet speed-time profile during a 15-min experiment.
- Fig. S44. Impact of number of droplet placed in the dish on the droplet speed-time profile during a 15-min experiment.
- Fig. S45. Same as fig. S44 with standard deviation.
- Fig. S46. Hydrodynamic diameter of micelles in the aqueous phase through time.
- Fig. S47. Conceptual design of the new Dropfactory laboratory robot.
- Fig. S48. Photo of the Dropfactory robot.
- Fig. S49. 3D view of the CAD design.
- Fig. S50. Geneva wheel design.
- Fig. S51. Geneva wheel top-plates design.
- Fig. S52. Photo of the Geneva wheel installed on Dropfactory.
- Fig. S53. Wheel stabilizer design and photo.
- Fig. S54. Modular linear actuator used designed for Dropfactory.
- Fig. S55. Photo of pumps and chemical inputs on Dropfactory.
- Fig. S56. Illustrating the working stations on the oil Geneva wheel.
- Fig. S57. Photo of the oil filling station.
- Fig. S58. Design of the oil filling head.
- Fig. S59. Photo of the oil stirring station with the small magnetic stirrer plate.
- Fig. S60. Photo and design of the oil cleaning station.
- Fig. S61. Photo of the oil drying stations.
- Fig. S62. Design of the drying station air guide.
- Fig. S63. Illustrating the working stations on the aqueous Geneva wheel.
- Fig. S64. Photo of the aqueous filling station.
- Fig. S65. Design of the aqueous filling station tube guide.
- Fig. S66. Photo of the syringe pick and place station.
- Fig. S67. Design of the modular syringe driver.
- Fig. S68. Photo of the recording station.
- Fig. S69. Design of the recording station.
- Fig. S70. Photo of the dish cleaning station.
- Fig. S71. Design of the dish cleaning station.
- Fig. S72. Photo of the drying station.
- Fig. S73. Visual display of droplet placement considered.
- Fig. S74. Comparison of biased droplet motion induced by droplet placement.
- Fig. S75. Droplet tracking via OpenCV.
- Fig. S76. Explanation of threshold definition for binarization of droplet video.
- Fig. S77. Image processing pipeline for the detection of droplet.
- Fig. S78. Visualization of the covered arena area metrics.
- Fig. S79. Visualization of the exploration metrics.
- Table S1. Description of each phases P1 to P6 with identifying criteria.
- Table S2. Parameters values for oil concentration curve fitting.
- Table S3. Measured PH of aqueous phase preparation.
- Legends for movies S1 to S6
- References (*34*–*56*)

Download PDF

**Other Supplementary Material for this manuscript includes the following:**

- Movie S1 (.mp4 format). Operation of the parallelized droplet robot.
- Movie S2 (.mp4 format). Progression of the exploration for each algorithm.
- Movie S3 (.mp4 format). 1st, 10th, and 50th highest speed droplet recipes from each algorithm.
- Movie S4 (.mp4 format). Effect of temperature on a droplet recipe during a 90s experiment.
- Movie S5 (.mp4 format). Effect of temperature on a droplet recipe during a 15-min experiment.
- Movie S6 (.mp4 format). Effect of temperature on the release of methylene-blue dye.

**Files in this Data Supplement:**

- Adobe PDF - aay4237\_SM.pdf
